# Genetic variation analysis of the Bali street dog using microsatellites

**DOI:** 10.1186/1471-2156-6-6

**Published:** 2005-02-08

**Authors:** Dawn N Irion, Alison L Schaffer, Sherry Grant, Alan N Wilton, Niels C Pedersen

**Affiliations:** 1 Veterinary Genetics Laboratory, Center for Veterinary Genetics, School of Veterinary Medicine, University of California, Davis, California 95616, USA; 2 Yayasan Yudisthira, Sanur, Bali 80228, Indonesia; 3 School of Biotechnology and Biomolecular Sciences, University of New South Wales, Sydney NSW 2052, Australia

## Abstract

**Background:**

Approximately 800,000 primarily feral dogs live on the small island of Bali. To analyze the genetic diversity in this population, forty samples were collected at random from dogs in the Denpasar, Bali region and tested using 31 polymorphic microsatellites. Australian dingoes and 28 American Kennel Club breeds were compared to the Bali Street Dog (BSD) for allelic diversity, heterozygosities, F-statistics, G_ST _estimates, Nei's DA distance and phylogenetic relationships.

**Results:**

The BSD proved to be the most heterogeneous, exhibiting 239 of the 366 total alleles observed across all groups and breeds and had an observed heterozygosity of 0.692. Thirteen private alleles were observed in the BSD with an additional three alleles observed only in the BSD and the Australian dingo. The BSD was related most closely to the Chow Chow with a F_ST _of 0.088 and also with high bootstrap support to the Australian dingo and Akita in the phylogenetic analysis.

**Conclusions:**

This preliminary study into the diversity and relationship of the BSD to other domestic and feral dog populations shows the BSD to be highly heterogeneous and related to populations of East Asian origin. These results indicate that a viable and diverse population of dogs existed on the island of Bali prior to its geographic isolation approximately 12,000 years ago and has been little influenced by domesticated European dogs since that time.

## Background

**Figure 1:**
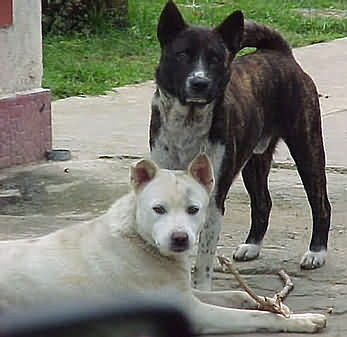
Typical Balinese street dogs. Their phenotypic appearance is similar to that described for randomly breeding feral dog subpopulations in other parts of the world.

Bali, a province of the Republic of Indonesia, is an island just 87 km from north to south and 142 km from east to west and home to more than 2.9 million people [1]. Approximately 800,000 stray dogs (Fig. 1) also live on the island based on a survey conducted by the Bali Street Dog Foundation (personal communication). Only a small percentage of these dogs live in homes or are provided routine veterinary care [2].

**Figure 2:**
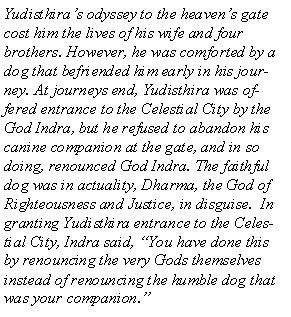
The Story of Yudisthira [4].

More than 90% of the residents of Bali are Hindu [3] with myth and ritual playing a vital part of daily life [1]. The dog is also an important part of Balinese life and mythology. A popular tale from the Mahabharata [4] describes King Yudisthira's journey to Heaven's Gate, and his love for a dog that befriended him on his arduous and tragic journey (Fig. 2). As a direct result of such mythology, BSDs are treated with a degree of reverence and are often provided ceremonial food offerings [2]. The deliberate killing of street dogs is not typically practiced, because Balinese people believe that all things should be allowed to die naturally [2]. These cultural mores have contributed to the current overpopulation of dogs on the island.

As a result of overpopulation, many BSDs suffer from chronic skin diseases, internal parasites, parvo- and distemper-virus infections, and malnutrition. In an effort to reduce the dog population and to care for their medical needs, the Bali Street Dog Foundation (Yayasan Yudisthira Swarga) was founded in 1998 [2]. They provide emergency care, treatment for skin disease and parasites, sterilization, public education on the plight of feral dogs, and improved veterinarian training. Twenty to 30 dogs are sterilized each day, with more than 9,000 dogs sterilized to date.

The BSD population is of interest for both its genetic diversity and historical relationships. It is also a population that has bred more or less randomly for thousands of years with limited genetic influx, due mainly to geographic barriers and a strict rabies control program in effect since 1926. The present study is concerned with the genetic diversity of this unique canine population and its relationship to other canine subpopulations in Asia and throughout the world. Data presented herein was derived from the DNA testing of 40 BSD samples from the Denpasar city region of Bali with 31 polymorphic microsatellite loci. The genetic diversity of the BSD was compared to that of the Australian dingo and 28 American Kennel Club (AKC) breeds.

## Results

### Locus diversity

Analysis of locus diversity across all 30 subpopulations revealed that the number of observed alleles ranged from six to 20 with a total of 366 for all loci (Table 1). Overall heterozygosity of the loci was high, with an average of 0.779, and all but four loci having H_T _values greater than 0.700. Average H_S _was 0.577 for the 30 subpopulations, with all but three loci having H_S _values greater than 0.500. The H_S _and H_T _values were closest for C23.123 and farthest for C22.279 and C10.404. HWE analysis revealed that all but one locus had at least one population out of equilibrium for the 30 populations sampled. C01.424, C31.646 and CPH16 had 7 populations out of HWE and AHT130 did not have any populations with p values below 0.05. The level of locus diversity attributable to subpopulation structure was evaluated with two statistics – R_ST _and F_ST_. Both statistics gave similar average values at 0.230 and 0.236 respectively. However, R_ST _ranged from 0.098 to 0.486 while F_ST _ranged from 0.179 to 0.328.

**Table 1:** Observed number of alleles, average total heterozygosity (H_T_), average subpopulation heterozygosity (H_S_), number of populations out of HWE, average p values, R_ST_, F_ST_, R_ST_/F_ST_ratio, G_ST_and pairwise F_ST_values for 31 loci.

										BSD Pairwise F_ST _by Locus	
	Chr.	Num. Observed Alleles	H_T_	H_S_	Num. Loci with p value <0.05	Average p value	R_ST_	F_ST_	R_ST_/F_ST_	× Dingo	× Chow
CPH16	CFA20	11	0.829	0.610	7	0.407	0.098	0.233	0.420	0.005	0.132
C08.618	CFA08	9	0.744	0.553	4	0.481	0.148	0.229	0.646	0.071	0.185
FH2001	CFA23	13	0.791	0.593	4	0.433	0.167	0.225	0.741	0.083	0.100
C20.446	CFA20	10	0.729	0.553	3	0.541	0.173	0.215	0.804	0.123	0.105
C01.424	CFA01	9	0.716	0.476	7	0.475	0.258	0.320	0.807	0.125	0.110
CPH02	CFA32	9	0.693	0.520	4	0.499	0.194	0.223	0.871	0.148	0.066
FH2004	CFA11	18	0.809	0.611	3	0.522	0.187	0.214	0.873	0.057	0.060
AHT137	CFA11	14	0.861	0.672	2	0.427	0.176	0.197	0.893	0.064	0.188
C03.877	CFA03	12	0.730	0.509	1	0.524	0.268	0.275	0.977	0.244	0.114
C06.636	CFA06	12	0.652	0.483	6	0.435	0.233	0.237	0.982	0.069	0.024
AHT121	CFA13	18	0.865	0.632	1	0.511	0.255	0.251	1.016	0.098	0.063
VIASD10	CFA07	9	0.759	0.555	2	0.525	0.249	0.233	1.066	0.268	0.030
C31.646	CFA31	14	0.814	0.566	7	0.410	0.301	0.281	1.075	0.088	0.036
RVC1	CFA15	9	0.774	0.569	4	0.458	0.270	0.242	1.119	0.355	0.216
LEI002	CFA27	11	0.737	0.551	4	0.534	0.259	0.229	1.127	0.107	0.149
LEI004	CFA37	13	0.667	0.509	4	0.510	0.246	0.219	1.128	0.133	0.051
C28.176	CFA28	10	0.735	0.546	6	0.400	0.270	0.234	1.152	0.249	0.013
C22.279	CFA22	11	0.836	0.529	2	0.441	0.262	0.214	1.226	0.201	0.109
PEZ02	Unlinked	12	0.762	0.600	1	0.567	0.232	0.187	1.242	0.134	0.027
FH2054	CFA12	10	0.848	0.654	4	0.524	0.251	0.199	1.261	0.055	0.069
C23.123	CFA23	8	0.766	0.636	4	0.402	0.351	0.278	1.263	0.110	0.002
CPH08	CFA19	11	0.765	0.582	4	0.465	0.284	0.222	1.276	0.082	0.056
C14.866	CFA14	10	0.840	0.604	3	0.473	0.330	0.255	1.293	0.180	0.112
PEZ08	CFA17	17	0.859	0.684	5	0.465	0.235	0.179	1.312	0.121	0.079
AHT130	CFA18	11	0.829	0.614	0	0.539	0.313	0.235	1.331	0.116	0.105
AHT111	CFA02	11	0.785	0.582	4	0.371	0.348	0.246	1.414	0.214	0.006
C10.404	CFA10	13	0.865	0.558	3	0.526	0.486	0.328	1.479	0.177	0.160
C09.250	CFA09	10	0.830	0.582	1	0.491	0.412	0.272	1.513	0.016	0.042
FH2140	CFA05	20	0.795	0.621	2	0.560	0.297	0.192	1.546	0.119	0.072
AHT139	CFA15	6	0.664	0.508	4	0.452	0.330	0.206	1.604	0.085	0.078
CPH03	CFA06	15	0.815	0.612	2	0.526	0.394	0.229	1.724	0.017	0.180
All		366	0.779	0.577	108	0.481	0.230	0.236	1.135	0.126	0.088

### Bali street dog diversity

Overall, the BSD was the most genetically diverse population surveyed here, displaying 239 total alleles out of the 366 seen in all 30 subpopulations or 65.3% of the total observed alleles (Table 2). The Australian dingo displayed 144 alleles and the AKC breeds displayed 138.8 on average. Analysis of expected (H_E_) and observed (H_O_) heterozygosities (Table 2) revealed that the BSD had a 44.0% higher H_E _than the Australian dingo (0.736 vs. 0.511) and a 28.4% higher H_E _than the average AKC breed (0.573). H_O _was also highest in the BSD at 0.692, versus 0.426 in the Australian dingo and 0.563 in the average AKC breed.

**Table 2:** Total number of alleles (N_A_) observed, range of the lowest and highest number of observed alleles per locus, expected heterozygosity (H_E_), observed heterozygosity (H_O_), F_IS_, number of loci out of HWE and average p values for all 31 loci for the BSD, a bootstrapped sampling of the BSD, the Australian dingo, the American Kennel Club breeds and for all subpopulations.

	N_A _Observed	N_A _Range	H_E_	H_O_	F_IS_	Num. Loci with p value <0.05	Average p value
Bali Street Dog	239	3 – 14	0.736	0.692	0.097	4	0.357
Bali Street Dog_20_	214.6	---	0.727	0.692	---	---	---
Australian dingo	144	2 – 9	0.511	0.426	0.194	12	0.284
AKC Breeds	138.8	2.2 – 7.8	0.573	0.563	0.137	3.29	0.492
All Populations	366	6 – 20	0.577	0.562	0.136	3.60	0.492

In order to evaluate the bias of sampling twice the number of BSDs, twenty samples were taken at random from the total pool of 40 for bootstrap determinations, and the number of observed alleles, H_E_, and H_O _were calculated. This process was repeated for 10,000 iterations and the average value for each measurement was determined. The average bootstrap value for the number of observed alleles for 20 BSDs was 214.6. The average bootstrapped H_E _and H_O _values for the BSD were 0.727 and 0.692 respectively.

To understand the loss of approximately 24 observed alleles after bootstrapping, the allele frequencies for the BSD at each locus was examined. While the BSD had the highest number of alleles, they also had the highest number of alleles with a frequency below 5% (67 out of 239, data not shown).

F_IS _estimates were calculated to assess the level of inbreeding for each subpopulation (Table 2). The BSD had the lowest value at 0.097 and the Australian dingo had the highest at 0.194 with the average of AKC breeds at 0.137.

### Private alleles

Allele frequency analysis also revealed that 10 private alleles were observed in the BSD as well as three alleles shared only with the Australian dingo (Table 3). The majority of private alleles in the BSD were below 5% in frequency with the exception of AHT121 where the private alleles had a combined frequency of 18.75%. The BSD and the Australian dingo also shared three alleles not seen in any AKC breed at locus C10.404 with a combined frequency of 16.25% in the BSD and 75% in the Australian dingo.

**Table 3:** Private alleles for the BSD and Australian dingo subpopulations relative to the 28 comparison AKC breeds.

Locus	Allele	Pop	Freq.	Pop	Freq.
AHT111	92	BSD	0.013		
AHT121	82	BSD	0.075		
AHT121	90	BSD	0.113		
C06.636	158	BSD	0.013		
C10.404	168	BSD	0.038	Dingo	0.075
C10.404	170	BSD	0.063	Dingo	0.450
C10.404	172	BSD	0.063	Dingo	0.225
C10.404	174	BSD	0.038		
C23.123	154	BSD	0.013		
FH2140	160	BSD	0.013		
FH2140	171	BSD	0.025		
PEZ02	144	BSD	0.013		
VIASD10	94	BSD	0.013		

### Asian alleles

Several additional unique alleles were found only in the BSD, Australian dingo, Chow Chow and Akita; demonstrating a closer relationship of the BSD to Asian versus non-Asian dogs (data not shown). Appearing at the highest frequency was allele 201 of locus CPH08 in the BSD, Australian dingo and Chow Chow at frequencies of 21.3%, 10% and 20%, respectively. Further, the BSD, Australian dingo, Chow Chow and Akita share allele 113 of locus C22.279 at frequencies of 23.8%, 67.5%, 15% and 5%, respectively. Results for locus PEZ08 demonstrate a lack of influence of European alleles where a high frequency of deviations from n+4 alleles were observed in the AKC breeds sampled yet no deviation from n+4 alleles was observed in the BSD or the Australian dingo.

A pairwise F_ST _analysis was performed for each locus between the BSD and the two closest subpopulations: the Australian dingo and Chow Chow (Table 1). The BSD was most similar to the Australian dingo at locus CPH16 with a F_ST _of 0.005 and to the Chow Chow at locus C23.123 with a F_ST _of 0.002. The BSD was most dissimilar to the Australian dingo and Chow Chow at locus RVC1 with a F_ST _of 0.355 and 0.216 respectively. Several loci had similar distances for both population pairs, such as locus C01.424 or locus C20.446 and may indicate areas of the genome that are neutral to either environmental or human selection.

### Genetic distance relationships

Further distance analysis was performed for all 31 loci between all 30 subpopulations using both Nei's DA distance and pairwise F_ST _estimates (Table 4). Across all loci, the BSD shared allele frequencies most closely with the Chow Chow (DA = 0.242, F_ST _= 0.088) and the Australian dingo (DA = 0.242, F_ST _= 0.126), and least closely with the Airedale Terrier (DA = 0.454, F_ST _= 0.258).

**Table 4:** Nei's DA distance (lower triangle) and mean F_ST_estimates (upper triangle) between each pair of 9 dog subpopulations represented graphically in Figure 3.

	*BSD*	*Dingo*	*Chow*	*Akita*	*AES*	*AS*	*AT*	*BCO*	*BLT*	*MBT*	*BMD*	*BS*	*BT*	*BU*	*BX*	*BZ*	*DP*	*GH*	*GR*	*JRT*	*KE*	*LR*	*NE*	*PG*	*PM*	*PPN*	*PWC*	*RR*	*WM*	*YT*
BSD		*0.13*	*0.09*	*0.13*	*0.14*	*0.14*	*0.26*	*0.19*	*0.26*	*0.25*	*0.21*	*0.15*	*0.17*	*0.18*	*0.33*	*0.18*	*0.26*	*0.17*	*0.14*	*0.11*	*0.17*	*0.17*	*0.18*	*0.23*	*0.13*	*0.11*	*0.15*	*0.15*	*0.16*	*0.13*
Dingo	0.24		*0.23*	*0.26*	*0.27*	*0.27*	*0.41*	*0.32*	*0.42*	*0.40*	*0.37*	*0.30*	*0.29*	*0.29*	*0.50*	*0.31*	*0.41*	*0.30*	*0.26*	*0.25*	*0.31*	*0.30*	*0.31*	*0.39*	*0.27*	*0.25*	*0.29*	*0.29*	*0.29*	*0.25*
Chow	0.24	0.40		*0.19*	*0.22*	*0.21*	*0.34*	*0.26*	*0.33*	*0.32*	*0.29*	*0.24*	*0.23*	*0.24*	*0.41*	*0.26*	*0.34*	*0.26*	*0.21*	*0.17*	*0.23*	*0.25*	*0.25*	*0.31*	*0.20*	*0.17*	*0.22*	*0.22*	*0.23*	*0.17*
Akita	0.29	0.41	0.36		*0.19*	*0.19*	*0.33*	*0.26*	*0.35*	*0.32*	*0.31*	*0.20*	*0.22*	*0.25*	*0.40*	*0.23*	*0.30*	*0.24*	*0.18*	*0.17*	*0.23*	*0.22*	*0.26*	*0.30*	*0.20*	*0.17*	*0.20*	*0.20*	*0.22*	*0.19*
AES	0.28	0.43	0.43	0.37		*0.10*	*0.29*	*0.16*	*0.33*	*0.30*	*0.23*	*0.14*	*0.16*	*0.19*	*0.32*	*0.24*	*0.24*	*0.13*	*0.12*	*0.10*	*0.14*	*0.16*	*0.18*	*0.25*	*0.12*	*0.10*	*0.17*	*0.13*	*0.16*	*0.13*
AS	0.27	0.42	0.38	0.36	0.20		*0.27*	*0.15*	*0.32*	*0.29*	*0.24*	*0.16*	*0.17*	*0.18*	*0.35*	*0.21*	*0.23*	*0.16*	*0.13*	*0.10*	*0.17*	*0.16*	*0.19*	*0.23*	*0.13*	*0.10*	*0.16*	*0.13*	*0.17*	*0.12*
AT	0.45	0.55	0.50	0.45	0.40	0.36		*0.35*	*0.46*	*0.44*	*0.37*	*0.34*	*0.32*	*0.34*	*0.50*	*0.31*	*0.46*	*0.32*	*0.29*	*0.26*	*0.34*	*0.32*	*0.32*	*0.42*	*0.27*	*0.28*	*0.34*	*0.32*	*0.31*	*0.28*
BCO	0.35	0.49	0.48	0.44	0.26	0.25	0.45		*0.39*	*0.37*	*0.24*	*0.21*	*0.21*	*0.25*	*0.41*	*0.29*	*0.30*	*0.22*	*0.21*	*0.15*	*0.24*	*0.23*	*0.25*	*0.30*	*0.17*	*0.16*	*0.20*	*0.22*	*0.21*	*0.16*
BLT	0.43	0.55	0.51	0.52	0.46	0.42	0.51	0.54		*0.10*	*0.43*	*0.35*	*0.40*	*0.35*	*0.49*	*0.40*	*0.44*	*0.36*	*0.30*	*0.25*	*0.34*	*0.32*	*0.35*	*0.46*	*0.33*	*0.27*	*0.36*	*0.36*	*0.36*	*0.30*
MBT	0.41	0.52	0.48	0.48	0.41	0.38	0.48	0.50	0.09		*0.54*	*0.52*	*0.54*	*0.38*	*0.46*	*0.50*	*0.52*	*0.48*	*0.43*	*0.36*	*0.42*	*0.41*	*0.34*	*0.44*	*0.30*	*0.26*	*0.35*	*0.34*	*0.33*	*0.29*
BMD	0.39	0.51	0.47	0.49	0.36	0.35	0.45	0.36	0.54	*0.42*		*0.23*	*0.28*	*0.30*	*0.42*	*0.35*	*0.36*	*0.28*	*0.23*	*0.18*	*0.29*	*0.25*	*0.28*	*0.37*	*0.21*	*0.20*	*0.26*	*0.24*	*0.26*	*0.21*
BS	0.32	0.46	0.45	0.38	0.28	0.28	0.48	0.35	0.52	*0.34*	0.36		*0.20*	*0.24*	*0.35*	*0.23*	*0.28*	*0.17*	*0.15*	*0.13*	*0.19*	*0.15*	*0.22*	*0.30*	*0.15*	*0.11*	*0.18*	*0.15*	*0.19*	*0.17*
BT	0.36	0.48	0.44	0.41	0.29	0.30	0.43	0.35	0.59	*0.37*	0.42	0.35		*0.23*	*0.40*	*0.24*	*0.28*	*0.19*	*0.20*	*0.16*	*0.22*	*0.24*	*0.23*	*0.28*	*0.17*	*0.18*	*0.22*	*0.18*	*0.24*	*0.17*
BU	0.31	0.41	0.42	0.42	0.29	0.27	0.41	0.39	0.42	*0.31*	0.41	0.40	0.36		*0.36*	*0.27*	*0.30*	*0.22*	*0.22*	*0.17*	*0.26*	*0.24*	*0.28*	*0.32*	*0.22*	*0.16*	*0.24*	*0.19*	*0.26*	*0.19*
BX	0.47	0.58	0.55	0.54	0.37	0.40	0.51	0.48	0.49	*0.46*	0.46	0.45	0.50	0.36		*0.44*	*0.44*	*0.40*	*0.33*	*0.31*	*0.39*	*0.39*	*0.40*	*0.46*	*0.35*	*0.31*	*0.40*	*0.36*	*0.40*	*0.31*
BZ	0.35	0.46	0.45	0.41	0.40	0.33	0.39	0.44	0.52	*0.37*	0.47	0.36	0.39	0.36	0.50		*0.32*	*0.21*	*0.23*	*0.17*	*0.27*	*0.23*	*0.28*	*0.34*	*0.20*	*0.19*	*0.24*	*0.25*	*0.27*	*0.20*
DP	0.45	0.57	0.55	0.48	0.39	0.37	0.60	0.44	0.57	*0.41*	0.47	0.44	0.43	0.41	0.48	0.46		*0.29*	*0.28*	*0.23*	*0.29*	*0.30*	*0.34*	*0.37*	*0.24*	*0.23*	*0.29*	*0.26*	*0.32*	*0.26*
GH	0.35	0.46	0.46	0.44	0.27	0.27	0.41	0.34	0.50	*0.35*	0.40	0.30	0.32	0.35	0.49	0.33	0.43		*0.19*	*0.16*	*0.21*	*0.21*	*0.23*	*0.28*	*0.17*	*0.15*	*0.22*	*0.15*	*0.23*	*0.16*
GR	0.33	0.43	0.42	0.39	0.25	0.26	0.37	0.33	0.43	*0.29*	0.37	0.31	0.35	0.35	0.40	0.37	0.44	0.34		*0.11*	*0.18*	*0.13*	*0.18*	*0.31*	*0.15*	*0.10*	*0.17*	*0.16*	*0.16*	*0.14*
JRT	0.25	0.43	0.32	0.34	0.21	0.20	0.37	0.27	0.38	*0.24*	0.30	0.28	0.30	0.27	0.39	0.31	0.38	0.29	0.25		*0.15*	*0.11*	*0.15*	*0.26*	*0.11*	*0.08*	*0.14*	*0.13*	*0.16*	*0.07*
KE	0.33	0.50	0.42	0.41	0.27	0.28	0.44	0.35	0.47	*0.32*	0.42	0.36	0.36	0.38	0.46	0.42	0.42	0.36	0.34	0.28		*0.22*	*0.25*	*0.32*	*0.15*	*0.15*	*0.22*	*0.17*	*0.23*	*0.16*
LR	0.33	0.45	0.46	0.39	0.29	0.28	0.42	0.36	0.42	*0.31*	0.36	0.26	0.38	0.37	0.46	0.35	0.44	0.32	0.26	0.24	0.37		*0.20*	*0.33*	*0.15*	*0.12*	*0.23*	*0.19*	*0.17*	*0.16*
NE	0.35	0.45	0.44	0.45	0.28	0.30	0.42	0.38	0.45	0.45	0.37	0.37	0.38	0.39	0.46	0.43	0.46	0.34	0.33	0.28	0.40	0.34		*0.36*	*0.19*	*0.17*	*0.21*	*0.21*	*0.22*	*0.20*
PG	0.39	0.54	0.48	0.45	0.38	0.35	0.51	0.44	0.60	0.56	0.50	0.45	0.43	0.44	0.52	0.51	0.52	0.43	0.49	0.41	0.48	0.46	0.51		*0.27*	*0.25*	*0.30*	*0.28*	*0.30*	*0.27*
PM	0.26	0.46	0.39	0.38	0.23	0.25	0.41	0.29	0.49	0.45	0.35	0.29	0.29	0.34	0.45	0.34	0.41	0.30	0.30	0.22	0.27	0.28	0.34	0.43		*0.11*	*0.19*	*0.16*	*0.14*	*0.13*
PPN	0.26	0.42	0.37	0.35	0.22	0.21	0.39	0.28	0.42	0.40	0.32	0.26	0.34	0.28	0.38	0.32	0.37	0.30	0.24	0.20	0.27	0.24	0.31	0.39	0.24		*0.15*	*0.13*	*0.13*	*0.11*
PWC	0.31	0.45	0.41	0.39	0.30	0.27	0.47	0.32	0.50	0.48	0.41	0.33	0.37	0.39	0.49	0.37	0.48	0.37	0.31	0.27	0.35	0.34	0.35	0.45	0.32	0.28		*0.17*	*0.21*	*0.17*
RR	0.28	0.43	0.40	0.37	0.25	0.22	0.39	0.33	0.49	0.45	0.36	0.29	0.27	0.29	0.45	0.38	0.41	0.27	0.29	0.26	0.27	0.30	0.32	0.38	0.28	0.24	0.31		*0.21*	*0.16*
WM	0.32	0.45	0.41	0.41	0.29	0.28	0.39	0.34	0.47	0.44	0.37	0.31	0.36	0.40	0.47	0.40	0.45	0.37	0.29	0.29	0.36	0.26	0.34	0.41	0.28	0.27	0.35	0.28		*0.19*
YT	0.30	0.44	0.37	0.41	0.29	0.22	0.40	0.31	0.44	0.42	0.35	0.31	0.32	0.31	0.40	0.32	0.40	0.29	0.30	0.18	0.30	0.29	0.34	0.43	0.26	0.25	0.31	0.29	0.32	

**Figure 3:**
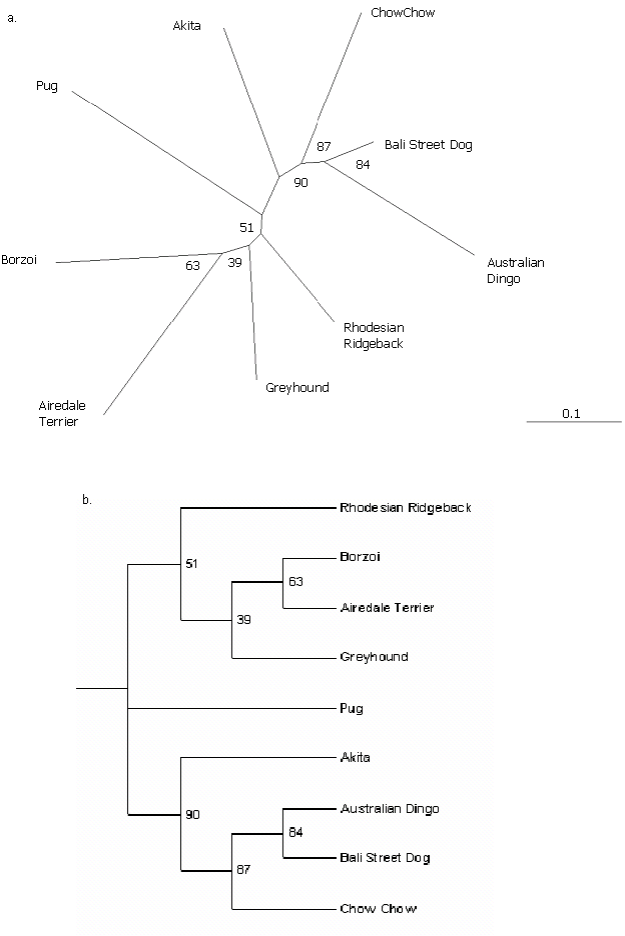
a. Unrooted neighbor-joining dendogram showing the genetic relationships among 9 dog subpopulations based on DA genetic distance. b. Rooted neighbor-joining dendogram showing the genetic relationships among 9 dog subpopulations based on DA genetic distance. In both versions of the dendogram the Pug did not cluster with any population but is placed intermediate between the Asian and non-Asian subpopulations.

Genetic distance relationships amongst the five Asian subpopulations were further explored using neighbor-joining dendograms with four non-Asian subpopulations for comparison (Fig. 3). The BSD, Chow Chow, Australian dingo and Akita clustered together in 90% of the trees. The BSD, Chow Chow and Australian dingo further clustered in 87% of the trees. The BSD and Australian dingo maintained their relationship within the larger cluster in 84% of the trees. In the remainder of the tree, the Rhodesian Ridgeback, Greyhound, Airedale Terrier and Borzoi maintained a relationship in 51% of the trees and the Airedale Terrier/Borzoi cluster was seen in 63% of the trees. The Pug did not maintain a relationship with any other breed in this analysis, but was intermediate to the Asian and non-Asian subpopulations.

## Discussion

### Population diversity

Microsatellites have been previously used to assess genetic diversity and relationships in feral dog subpopulations [6,7]. Kim et al. [6] found that H_O _was high in three feral dog subpopulations of Korea, Sakhalin and Taiwan, ranging from 0.539 in the Taiwanese to 0.717 in the Korean dogs. Given that the loci used in that study had an average allele number of 7.75, these values are similar to the H_O _of 0.692 observed in the BSD. Wilton et al. [7] surveyed a population of Australian dingoes and found an average H_O _of 0.387 using microsatellites with an average allele number of 6.93, similar to the H_O _of 0.426 for the Australian dingoes reported herein with an average allele number of 11.8.

Given the size of the island of Bali, it is extraordinary that 800,000 feral dogs can thrive and maintain such high levels of genetic diversity. Of all the subpopulations surveyed here, the BSD has the highest number of observed alleles, the highest heterozygosity, the fewest number of loci out of Hardy-Weinberg equilibrium and the lowest F_IS_. Even after adjusting for sample size, the BSD maintains their status as the most heterogeneous population in the study. Unlike the Australian dingo which exhibits a much lower level of diversity, the BSD findings suggest either a large founding population on Bali and/or a consistent genetic influx since the geographic isolation of ~12,000 years ago. This data also supports that the BSD appears to approximate a randomly breeding population with little selection pressure.

When comparing the heterogeneity of the BSD to that observed within the AKC breeds some caveats should be addressed. One may initially expect long established, well-defined dog breeds to be much less heterogeneous than reported here. While some breeds do have a low H_E_, such as the Boxer with a H_E _of 0.320, breeds like the Jack Russell Terrier have a high H_E _of 0.713 and overall their H_E _is higher than that of the dingo. Of first note, the selection of the dogs that contribute to a breed composition mostly occurs prior to official breed recognition primarily by genetic drift due to geographic isolation and selection for particular working or physical characteristics. After official breed recognition future breeding choices are based primarily on the availability of sires and dams that approximate the breed standard. As a result, there is a founding population that proceeds to breed mostly by convenience. Also, many breed standards have changed considerably over the years resulting in retention of a certain level of diversity within each breed, some breeds retaining much more than others. Finally, dogs comprising the comparison AKC breeds were sampled from across the United States, removing any geographical bias of the genotypes observed and slightly elevating the heterozygosities.

### Locus diversity

The average allelic diversity of the loci used in the present study was 11.8 alleles per locus, versus 7.75 in the Kim et al work [6]. However, the average number of alleles observed is 4.6 among the subpopulations in the present study and the average H_T _is 0.577. The average values for the 11 subpopulations surveyed in the Kim et al [6] work were 4.34 and 0.547, respectively. The higher total allelic diversity in the present study is likely due to the fact that nearly three times more subpopulations were studied.

R_ST _and F_ST _values were nearly identical across all subpopulations and all loci, indicating that approximately 23% of the differences observed in allele frequencies can be attributed to differences between subpopulations. F_ST _provides an unbiased estimate of genetic drift between subpopulations by comparing alleles identical by state. R_ST _takes advantage of the stepwise mutation model, which assumes that mutations most often occur as whole repeat unit losses or gains from the original allele size. As a result, the number of mutations provides an estimate of time from divergence. It is interesting, therefore, to compare R_ST _and F_ST _values by locus. Eighteen of the 31 loci studied have an R_ST _to F_ST _ratio greater than 1.1 (Table 1) indicating that the populations have been separated for a sufficient amount of time for mutations to impact genetic structure. An interesting exception is observed at CPH16 where the ratio is 0.420. CPH16 may have a mutation pattern where both stepwise additions and subtractions occur at equal and high frequency. Of note, the average pairwise R_ST _value between the BSD and each of the 29 comparison subpopulations is 0.056 at locus CPH16. The highest R_ST _to F_ST _ratio occurs at locus CPH03 with a value of 1.724. Interestingly, the BSD and the Australian dingo have a pairwise R_ST _value of 0.017 at CPH03, whereas the average value of the BSD compared to the other 28 subpopulations has a value of 0.254. The distance between the BSD and the Australian dingo at CPH03 may support that those two populations were isolated most recently from each other relative to the other 28 subpopulations.

### Bali street dog origin

The origin of the people of Bali is clouded by myth and a scarcity of archeological findings. Therefore, the origin of the dog on Bali is also speculative. Nonetheless, a hypothesis can be formed based on known human and dog histories. Current evidence points to an early migration of humans from Africa through Indonesia and into Australia approximately 60,000 to 70,000 years ago [8,9]. Recent excavations have also revealed that there was a great expansion into Indonesia from China between 4,000 and 5,000 years ago that could have contributed to a population pre-existing on Bali [1]. Supportive evidence that Indonesia was populated prior to 5,000 years ago is a higher degree of heterogeneity in the Indonesian population than seen in the North Asian population, suggesting that the Indonesia was populated earlier than regions to the North [10]. The "Slow Boat Model" for the peopling of Polynesia also suggests a prolonged mixing of Southeast Asians with Indonesians, which predated migration to the East [11]. In short, Indonesia appears to be a human genetic melting pot with genetic influences over tens of thousands of years.

**Figure 4:**
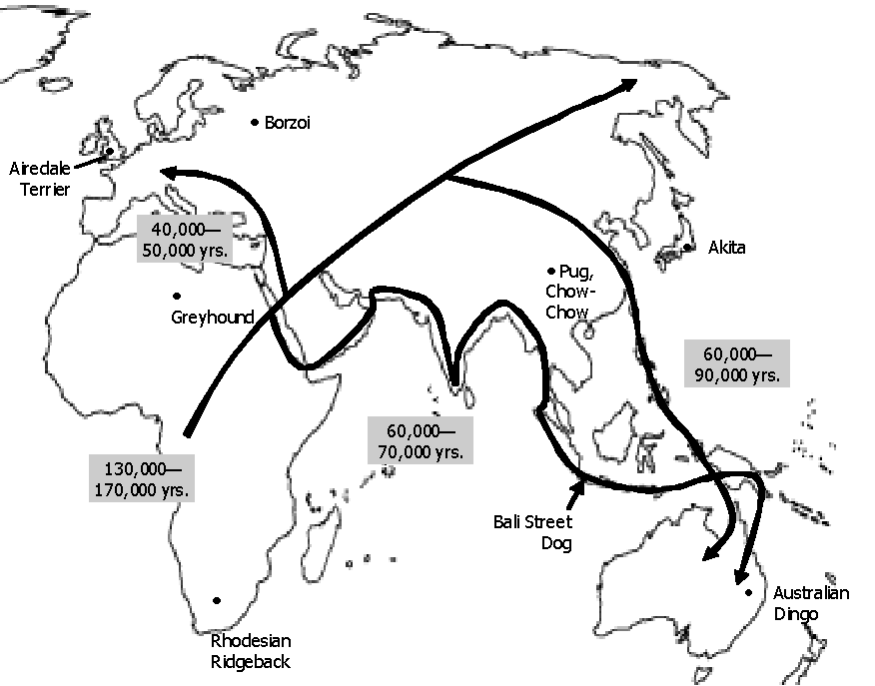
Human migration patterns proposed in "Tracing the road down under" [8], a summary of the *Modern human origins: Australian perspectives *conference at the University of New South Wales, September 2003 with locations of origin for 5 Asian and 4 non-Asian dog subpopulations.

The dog on the island of Bali may also be a parallel "canine genetic melting pot." While the domestication date of the dog is in much dispute [12], approximately 14,000 years ago is accepted as a late date. During the earliest human migrations through Indonesia however, it is highly possible that wolf packs or feral dogs traveled the same routes, establishing a feral population on Bali in the process. Even if humans were not capable of taming the dog at that time, dogs could still have benefited from close proximity to humans. Figure 4 shows a superimposition of the proposed geographic origin for five Asian and four non-Asian dog subpopulations presented herein and the major theorized human migration routes. It is noteworthy that the BSD, Chow Chow and Australian dingo, related breeds by genetic analysis, all share one proposed human migration route.

If a feral dog population was established on the island of Bali more than 14,000 years ago, then that population became isolated approximately 10,000 years ago when the sea levels drastically rose, submerging the land bridges of the Indonesia archipelago [13]. Geographic isolation was unlikely to have been absolute; genetic diversity of the BSD was invariably enhanced at various times by the influx of new dogs. At the time humans migrated to Indonesia from China, dogs were known to be domesticated and undoubtedly accompanied people as companions [17]. Mitochondrial DNA sequencing evidence suggests that the dingo was introduced into Australia about that time from the Indonesian archipelago [15,8,9]. Bali's documented history of repeated war and trade spanning the last 2,000 years [1,16,17] represents actions that are often associated with the introduction of new animals. Indeed, a somewhat free movement of dogs probably occurred up to 1926, when the import of dogs to Bali was greatly curtailed as a means to prevent the introduction of rabies [5]. This policy greatly reduced, though not eliminated, new outside introductions of new dogs to the island. In contrast to the Australian dingo population, which appears to have undergone a severe population bottleneck or founder effect based on microsatellite alleles and mtDNA [18], the BSD population maintains a high level of genetic variation. There is no evidence for a genetic bottleneck or small founding population for the BSD.

The relatedness of the BSD to the Australian dingo and the Chow Chow is evidenced by common unique alleles and allele frequencies despite the very different levels of genetic diversity between the subpopulations. According to the hypothesis presented herein, one could imagine that feral dog subpopulations were established throughout Indonesia with much mixing until ~12,000 years ago. At that time, each population became closed with little influx of new genetic material until humans migrated south from Asia between 4,000 and 5,000 years ago. The degree of influx since that period would have been influenced by the frequency of trade and conflict, factors determined by accessibility, available natural resources, and political structure. The island of Bali is historically a less visited island than it's neighbor Java and therefore the indigenous dog population would have been subjected to less influence.

## Conclusions

This study into the diversity and relationship of the BSD to other domestic and feral dog populations shows the BSD to be highly diverse and related to populations of East Asian origin. These results indicate that a viable and diverse population of dogs existed on the island of Bali prior to its geographic isolation approximately 12,000 years ago and has been little influenced by domesticated European dogs since that time. It would be of interest to study feral subpopulations on other islands in the archipelago to determine if the same level of diversity is observed elsewhere, or if the situation on Bali is truly unique. Y-chromosome, mitochondrial and MHC marker typing on the BSD, as well as feral dogs from other regions, would help to determine if indeed dogs followed the same migration routes as their likely human companions.

## Methods

### Animal selection

BSDs were randomly captured and taken to a BSD Foundation field clinic for treatment or sterilization and simultaneously sampled for DNA collection with buccal swabs. Familial relationships of the BSDs sampled could not be easily determined; therefore the sample population was doubled (40 vs. 19–20 samples) over that of other study groups. Blood samples from the Australian dingo were taken from captive animals in Australia. Australian dingoes were known to be unrelated by at least one generation.

Dogs from 28 American Kennel Club (AKC) breeds, equally representing the AKC group designations, were sampled with buccal swabs for a previous study [19]. Twenty dogs were tested for each breed, with the exception of two breeds (Doberman Pinscher and the Border Collie) that comprised 19 individuals. The 28 breeds included were: Airedale Terrier, Akita, American Eskimo, Australian Shepherd, Belgian Tervuren, Bernese Mountain Dog, Border Collie, Borzoi, Boxer, Brittany, Bull Terrier, Bulldog, Chow Chow, Doberman Pinscher, Golden Retriever, Greyhound, Jack Russell Terrier, Keeshond, Labrador Retriever, Miniature Bull Terrier, Norwegian Elkhound, Papillon, Pembroke Welsh Corgi, Pomeranian, Pug, Rhodesian Ridgeback, Weimaraner, and Yorkshire Terrier. Dogs within each breed were unrelated by at least one generation.

### Marker selection

Thirty-one of the 100 microsatellites multiplexed into 12 PCRs by the Veterinary Genetics Laboratory [20] had been previously used to evaluate the Australian dingo samples (unpublished data). For comparison purposes, those same 31 microsatellites were selected for use in the present study. All markers but one (PEZ02) were mapped on either the 1999 canine genetic linkage map [21] or the Radiation hybrid map [22]. Loci selected for study represented 25 of the 38 autosomes of the dog, with five autosomes represented by two loci. The average distance for the markers on chromosomes CFA06, CFA11, CFA20 and CFA23 is 23.5 cM and 23.4 Mb between AHT139 and RVC1 on CFA15. As a result, only 25 loci are known to be unlinked. PEZ02 has not been mapped and may be linked to a marker in the study.

Forward primers were synthesized and dye labeled with either Fam, Hex or Vic, or Tamra or Ned (Applied Biosystems, Inc. (ABI), Foster City, CA). Reverse primers were synthesized by Operon (Alameda, CA). Primer sequences and concentrations for all markers are available as Additional file 1.

### Sample preparation and PCR conditions

BSD and AKC breed DNA was derived from buccal cells harvested from the inside of the cheek with nylon bristle cytology brushes (Medical Packaging Corp., Camarillo, CA). Samples were collected by owners or field volunteers and submitted directly to the laboratory. DNA was extracted by heating a single swab for 10 min at 95°C in 400 μl 50 mM NaOH and then neutralized with 140 μl 1 M Tris-HCl, pH 8.0. Australian dingo DNA was extracted from blood using a standard sodium hydroxide digest.

A 2 μl aliquot of extract was used in each PCR which equates to approximately 50 ng DNA. All markers and DNAs were amplified with a PCR reagent mix of 1X PCR buffer (ABI), 4.17 mM MgCl_2_, 200 μM of each dNTP (Hoffmann-La Roche Inc, Nutley, NJ), 0.6 unit AmpliTaq (ABI), and 2% DMSO (Sigma) then covered with 15 ul Chill-out™ Liquid Wax (MJ Research, Inc., Waltham, MA) to prevent evaporation. One of five thermal cycler programs was used for each primer mix ranging from 56° to 64° degrees for the annealing temperature. All PCR work was done in polycarbonate 96-well v-bottom microtiter plates (USA Scientific, Ocala, FL) on MJ Research PTC-100 thermal cyclers (MJ Research, Inc., Waltham, MA). Protocols are also available in Additional 1.

### Gel electrophoresis conditions and DNA fragment analysis

One μl aliquots of PCR product were mixed with 2 μl Fluorescent Ladder (CXR) 60–400 (Promega 400) or Internal Lane Standard 600 (Promega 600) (Promega, Madison, WI) fluorescent size standard, denatured on MJ Research PTC-100 thermal cyclers for three minutes at 95°C, then held at 5°C or placed on ice for at least one minute before gel loading. Two μl aliquots were then loaded onto a 6% denaturing polyacrylamide gel and run on an ABI 377 Automated Sequencer using ABI 10" × 7 1/8" short plates (12 cm). Gels were run at 1.10 kV (constant) voltage, 60.0 mA current, 200 W power, 51°C and 40.0 mW (constant) laser power for up to 2 hours when using Promega 400, and up to 3 hours using Promega 600. DNA fragment analysis was performed with in-house designed STRand software [23], which replaces ABI Genotyper and Genescan software. This data was then transferred to an in-house database compatible with the STRand software.

### Statistical analysis

Allelic diversity and observed heterozygosities (H_O_) were determined by direct counting for each of the 30 subpopulations. Hardy-Weinberg equilibrium (HWE) tests were performed using Genepop version 3.4 [24]. Pairwise F_ST _estimates and subpopulation expected heterozygosities (H_E_) for the 30 breeds or dog groups were performed using Genepop version 3.4 [24]. F_IS _estimates (inbreeding coefficient of each subpopulation) for each allele following Weir and Cockerham [25] were calculated using Genepop version 3.4 and are presented as averages across all loci.

Gene diversity or total population heterozygosity (H_T_) and its associated parameters, H_S _(average heterozygosity among subpopulations) and G_ST _(coefficient of genetic differentiation), were calculated across all loci using the public domain software, DISPAN [26]. Two additional measures of variance, F_ST _[25] and R_ST _[27,28] were calculated using Genepop version 3.4. A pairwise genetic distance matrix using Nei's DA distance was also created using DISPAN with bootstrapping. Genotype data for all populations is available in Additional file 2.

### Phylogenetic tree construction

Allele frequencies were used to compute a matrix of genetic distances [29], which were then used to construct a phylogenetic tree of relationships among 5 Asian and 4 non-Asian dog subpopulations. Takezaki's [30] POPTREE program was used to create a neighbor joining tree using DA distances with 1000 bootstrap replications. The output of POPTREE was then converted to the New Hampshire format for editing in the stand alone program TREEVIEW version 1.6.6 [31] and bootstrap values were added.

## Abbreviations

BSD: Bali Street Dog

F_IS_, F_ST _R_ST_, G_ST_: F-statistics indices

H_S_, H_T_, H_E_, H_O_: Heterozygosity indices

HWE: Hardy-Weinberg equilibrium

N_A_: Number of alleles

AES: American Eskimo Dog

AS: Australian Shepherd

AT: Airedale Terrier

BCO: Border Collie

BLT: Bull Terrier

BMD: Bernese Mountain Dog

BS: Brittany Spaniel

BT: Belgian Tervuren

BU: Bulldog

BX: Boxer

BZ: Borzoi

Chow: Chow Chow

Dingo: Australian dingo

DP: Doberman Pinscher

GH: Greyhound

MBT: Miniature Bull Terrier

PG: Pug

RR: Rhodesian Ridgeback

GR: Golden Retriever

JRT: Jack Russell Terrier

KE: Keeshond

LR: Labrador Retriever

NE: Norwegian Elkhound

PG: Pug

PM: Pomeranian

PPN: Papillon

PWC: Pembroke Welsh Corgi

RR: Rhodesian Ridgeback

WM: Weimaraner

YT: Yorkshire Terrier

## Authors' contributions

DNI performed the majority of data acquisition and analysis, wrote first draft of the manuscript and prepared the final draft for submission. ALS performed the majority of sample processing, assisted in data acquisition and the writing of the subsequent drafts of the manuscript as well as final draft preparation. SG sampled the dogs tested, provided background for the manuscript and assisted in the final draft preparation. ANW provided the Australian dingo data for comparison and assisted in the subsequent drafts of the manuscript. NCP directed the research and assisted in the writing of the manuscript. All authors read and approved the final manuscript.

## Supplementary Material

Additional File 1 "MS 2784776144318916 Supplement1.xls" and contains the primer sequences, expected size range, primer concentration used and annealing temperatures used. In a separate sheet within the same file the protocols for each PCR reaction are listed. Click here for file

Additional File 2 "MS 2784776144318916 Supplement2.xls" and contains the individual genotype data for each animal used to derive the statistics and phylogenetic results presented herein. Click here for file

## References

[B1] Hobart A, Ramseyer U, Leemann A (1996). The peoples of Bali.

[B2] Yayasan Yudisthira Swarga (Bali Street Dog Foundation). http://www.yamp.com/balidogs/.

[B3] Vishva Hindu Parishad. http://www.vhp.org/englishsite/d.Dimensions_of_VHP/qVishwa%20Samanvya/vishvahinduparishadabroad.htm.

[B4] Buck W (1973). Mahabhrata.

[B5] Department of Foreign Affairs of the Republic of Indonesia. State Gazette 1912, No 452 and Ministry of Agriculture Decree No 892/Kpts/The Nation56/1997.

[B6] Kim KS, Tanabe Y, Park CK, Ha JH (2001). Genetic variability in East Asian dogs using microsatellite loci analysis. Journal of Heredity.

[B7] Wilton AN, Steward DJ, Zafiris K (1999). Microsatellite variation in the Australian dingo. Journal of Heredity.

[B8] Dayton L (2003). Modern human origins meeting: "Tracing the road down under". Science.

[B9] Dayton L (2003). Modern human origins meeting: "On the trail of the first Australian dingo". Science.

[B10] Faradz SM, Pattiiha MZ, Leigh DA, Jenkins M, Leggo J, Buckley MF, Holden JJ (2000). Genetic diversity at the FMR1 locus in the Indonesian population. Annals of Human Genetics.

[B11] Gibbons A (2001). The peopling of the Pacific. Science.

[B12] Pennisi E (2002). Canine evolution: A shaggy dog history. Science.

[B13] Hanebuth T, Stattegger K, Grootes PM (2000). Rapid flooding of the sunda shelf: A late-glacial sea-level record. Science.

[B14] Savolainen P, Zhang YP, Luo J, Lundeberg J, Leitner T (2002). Genetic evidence for an East Asian origin of domestic dogs. Science.

[B15] Savolainen P, Leitner T, Wilton AN, Matisoo-Smith E, Lundeberg J (2004). A detailed picture of the Origin of the Australian dingo, obtained from the study of Mitochondrial DNA. Proceedings of the National Academy of Sciences of the USA.

[B16] Ardika IW (1991). Archaeological research in northeastern Bali, Indonesia. PhD thesis.

[B17] Hartaningsih N, Dharma DMN, Rudiyanto MD (1999). Anjing Bali: Pemuliabiakan dan pelestarian (Bali Street Dogs: breeding and conservation).

[B18] Wilton AN, Dickman CR, Lunney D (2001). DNA Methods of Assessing Australian dingo Purity. A Symposium on the Australian dingo.

[B19] Irion DN, Schaffer AL, Famula TR, Eggleston ML, Hughes SS, Pedersen NC (2003). Analysis of genetic variation in 28 dog breed subpopulations with 100 microsatellite markers. Journal of Heredity.

[B20] Eggleston ML, Irion DN, Schaffer AL, Hughes SS, Draper JE, Robertson KR, Millon LV, Pedersen NC (2002). PCR multiplexed microsatellite panels to expedite canine genetic disease linkage analysis. Animal Biotechnology.

[B21] Neff MW, Broman KW, Mellersh CS, Ray K, Acland GM, Aguirre GD, Ziegle JS, Ostrander EA, Rine J (1999). A second-generation genetic linkage map of the domestic dog, Canis familiaris. Genetics.

[B22] Guyon R, Lorentzen TD, Hitte C, Kim L, Cadieu E, Parker HG, Quignon P, Lowe JK, Renier C, Gelfenbeyn B, Vignaux F, DeFrance HB, Gloux S, Mahairas GG, Andre C, Galibert F, Ostrander EA (2003). A 1-Mb Resolution Radiation Hybrid Map of the Canine Genome. Proceedings of the National Academy of Sciences of the USA.

[B23] STRand Nucleic Acid Analysis Software. http://www.vgl.ucdavis.edu/STRand.

[B24] Raymond M, Rousset F (1995). GENEPOP (version 1.2): population genetics software for exact tests and ecumenicism. Journal of Heredity.

[B25] Weir BS, Cockerham CC (1984). Estimating F-statistics for the analysis of population structure. Evolution.

[B26] Ota T (1993). Program DISPAN: Genetic distance and phylogenetic analysis.

[B27] Rousset F (1996). Equilibrium values of measure of population subdivision for stepwise mutation processes. Genetics.

[B28] Michalakis Y, Excoffier L (1996). A generic estimation of population subdivision using distances between alleles with special interest to microsatellite loci. Genetics.

[B29] Nei M (1987). Molecular evolutionary genetics.

[B30] Takezaki N POPTREE version for DOS. http://www.bio.psu.edu/People/Faculty/Nei/Lab/Programs.html.

[B31] Page RDM (2001). TreeView version 1.6.6. Distributed by the Division of Environmental and Evolutionary Biology, Institute of Biomedical and Life Sciences.

